# Is intracranial volume a risk factor for *IDH*-mutant low-grade glioma? A case–control study

**DOI:** 10.1007/s11060-022-04120-6

**Published:** 2022-08-27

**Authors:** Lisa Millgård Sagberg, Even Hovig Fyllingen, Tor Ivar Hansen, Per Sveino Strand, Aril Løge Håvik, Terje Sundstrøm, Alba Corell, Asgeir Store Jakola, Øyvind Salvesen, Ole Solheim

**Affiliations:** 1 Department of Neurosurgery, St Olavs Hospital, Trondheim University Hospital, Trondheim, Norway; 2 Department of Public Health and Nursing, Norwegian University of Science and Technology, Trondheim, Norway; 3 Department of Radiology and Nuclear Medicine, St Olavs Hospital, Trondheim University Hospital, Trondheim, Norway; 4 Department of Circulation and Medical Imaging, Norwegian University of Science and Technology, Trondheim, Norway; 5 Department of Neuromedicine and Movement Science, Norwegian University of Science and Technology, Trondheim, Norway; 6 Department of Physical Medicine and Rehabilitation, St Olavs Hospital, Trondheim University Hospital, Trondheim, Norway; 7 Department of Clinical Medicine, University of Bergen, Bergen, Norway; 8 Department of Neurology, Molde Hospital, Molde, Norway; 9 Department of Neurosurgery, Haukeland University Hospital, Bergen, Norway; 10 Department of Neurosurgery, Sahlgrenska University Hospital, Gothenburg, Sweden; 11 Institute of Neuroscience and Physiology, Department of Clinical Neuroscience, University of Gothenburg, Sahlgrenska Academy, Gothenburg, Sweden; 12 Clinical Research Unit, Department of Clinical and Molecular Medicine, Norwegian University of Science and Technology, Trondheim, Norway

**Keywords:** Brain neoplasms, Risk factors, Magnetic resonance imaging, Low-grade glioma, HUNT study

## Abstract

**Purpose:**

Risk of cancer has been associated with body or organ size in several studies. We sought to investigate the relationship between intracranial volume (ICV) (as a proxy for lifetime maximum brain size) and risk of *IDH*-mutant low-grade glioma.

**Methods:**

In a multicenter case–control study based on population-based data, we included 154 patients with *IDH*-mutant WHO grade 2 glioma and 995 healthy controls. ICV in both groups was calculated from 3D MRI brain scans using an automated reverse brain mask method, and then compared using a binomial logistic regression model.

**Results:**

We found a non-linear association between ICV and risk of glioma with increasing risk above and below a threshold of 1394 ml (*p* < 0.001). After adjusting for ICV, sex was not a risk factor for glioma.

**Conclusion:**

Intracranial volume may be a risk factor for IDH-mutant low-grade glioma, but the relationship seems to be non-linear with increased risk both above and below a threshold in intracranial volume.

## Introduction

Known risk factors for developing glioma include age, male sex, ionizing radiation, non-Hispanic white ethnicity, some specific germline variants, and a few rare hereditary syndromes. There also seems to be an inverse relationship between risk of glioma and atopic diseases [1]. Since risk of glioma is about 1.3–1.6 times higher in men than in women [2], and men have larger brain volumes and about 40% more glial cells [3], it could be speculated that brain size, perhaps associated with the number of stem cell divisions needed to maintain the glial volume, may be a risk factor. Indeed, we previously observed a strong association between intracranial volume (ICV) and risk of high-grade glioma [4]. However, incidence, age distribution and sex ratio of low-grade gliomas are different from high-grade gliomas [5, 6], and whether there is also an association between ICV and risk of low-grade gliomas is still not studied.

In the present study, we sought to compare ICV in patients with *IDH*-mutant low-grade glioma to healthy controls to investigate the possible relationship between ICV (as a proxy for lifetime maximum brain size) and the risk of the disease. Further, we explored if potential associations between ICV and *IDH*-mutant low-grade glioma was dependent on sex and molecular subtypes.

## Material and methods

### Study design and study population

In this multicenter case–control study, we included consecutive patients aged ≥ 18 years with primary *IDH*-mutant WHO grade 2 glioma. The patients were recruited from three different neurosurgical centers that each serve a defined catchment region: (a) Sahlgrenska University Hospital (Göteborg, Sweden) from 2010 to 2018, (b) Haukeland University Hospital (Bergen, Norway) from 2013 to 2017, and (c) St. Olavs Hospital (Trondheim, Norway) from 2007 to 2020. Together, these departments serve a population of about 3,5 million inhabitants. Exclusion criteria were infratentorial tumor location, Chiari malformation, previous intracranial surgery, and missing three-dimensional (3D) MRI scans. The Trøndelag Health Study (The HUNT study) MRI cohort from the general Norwegian population was used as a control group [7]. This is a database of 1006 healthy volunteers aged 50–66 years [8].

### Histopathology

*IDH*-status was determined by a neuropathologist using immunohistochemistry, fluorescence in situ hybridization (FISH), or multiplex ligation-dependent probe amplification (MLPA) methods, while 1p19q co-deletion was based on FISH, MLPA or from methylation array with analysis of copy number variation. In accordance with the 2021 WHO classification, we classified *IDH*-mutant tumors with 1p19q co-deletion as oligodendrogliomas and *IDH*-mutant tumors without 1p19q co-deletion as astrocytomas [9]. *IDH*-mutant tumors with missing 1p19q status were classified as non-specified diffuse gliomas. One patient with 1p19q co-deletion and missing *IDH-*status was also included (as oligodendroglioma).

### Intracranial tumor volume (ICV) calculations

An automated reverse brain mask method was used for ICV-calculations [10], where the method and workflow are described in a previous publication [4]. The earliest available 3D MRI scan (either diagnostic or preoperative) in each patient was used in the segmentation process of cases. Gadolinium contrast-enhanced T1-weighted 3D images were used in 138 cases, non-contrast enhanced T1-weighted images were used in 14 cases, while non-contrast enhanced T2-weighted images and T2 FLAIR images were used in one case each. The images were obtained using either a 1.5 or 3 Tesla scanner. All segmentations were visually quality assured by two of the authors (L.M.S and E.H.F). Minor segmentation revisions were performed by author E.H.F in two cases, where the automatic algorithm had excluded small parts of the ICV where tumors were adjacent to the dura. These segmentation volumes were only corrected in the tumor area to avoid bias by making manual changes to the segmentation volume in other areas. Segmentation corrections were performed in ITK-Snap version 3.6.0 for Mac [11]. ICV calculations in the control group were done in a previous study using the same automatic method [10].

### Statistical analyses

Data are presented as means with standard deviations (SD) or medians with range. Differences between groups were compared using two-samples Kolmogorov–Smirnov test or independent samples *t*-test, as appropriate. To explore the possible association between ICV and risk of *IDH*-mutant low-grade glioma, binomial logistic regression analyses were initially performed with glioma as the dependent variable, and ICV (continuous) and sex (categorical) as covariates. However, assumptions for binary logistic regression were not met due to non-linearity of the continuous ICV variable with respect to the logit of the dependent variable as assessed by the Box-Tidwell procedure. Therefore, a logarithmic transformation of the ICV variable was added to the regression model, improving model fit. Likelihood-ratio tests were used to compare regression models with and without sex as a covariate, including interactions between sex and ICV, and sex and logarithm of ICV. Goodness-of-fit in the final model was assessed using the Hosmer–Lemeshow test. Statistical significance level was set to *p* ≤ 0.05. Data analyses were performed using SPSS Statistics version 27 for Mac (IBM, Armonk, New York) and Stata version 17.0 for Mac (StataCorp LLC, College Station, Texas).

## Results

**Fig. 1:**
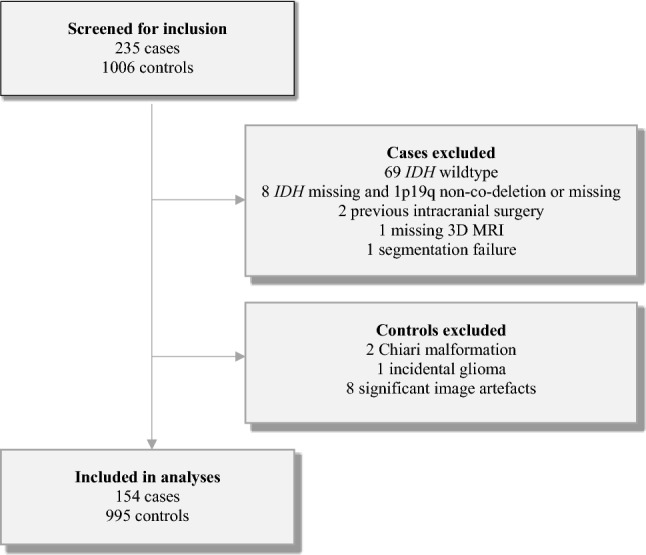
Flow chart of the inclusion process

A flow chart of the inclusion process is presented in Fig. 1. In total, 154 cases with primary *IDH*-mutant grade 2 gliomas were finally included (N = 71, 27, and 56 from hospital a, b, and c, respectively). Median age was 59 years (range 50–67 years) in the normal population (i.e., controls), and 40 years (range 18–77 years) in the low-grade glioma population (i.e., cases).

**Fig. 2:**
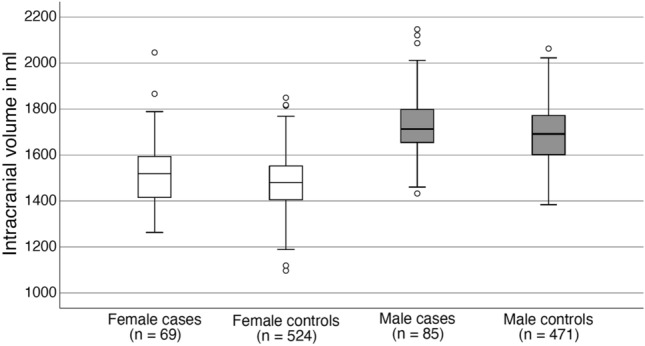
Distributions of intracranial volumes in cases and controls

In Fig. 2, boxplots showing the group distributions of ICV are presented separately for females and males. The group distributions were statistically significantly different in females (*p* = 0.026), while there was only a trend in males (*p* = 0.092).

**Fig. 3:**
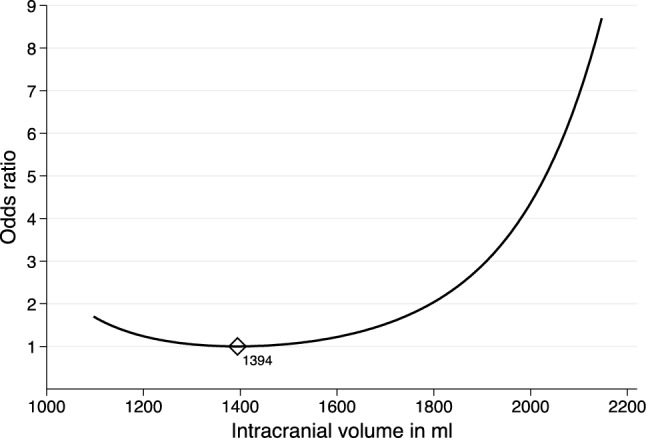
Odds ratio of IDH-mutant low-grade glioma based on ICV. Graph is presented within the upper and lower range limits for ICV. The diamond shaped marker illustrates the ICV value of lowest risk

Binomial logistic regression models with and without sex as a covariate were not statistically significantly different (*p* = 0.172), implying that risk of *IDH*-mutant low-grade glioma was not associated with sex after adjusting for ICV. The final binomial logistic regression model therefore included ICV and logarithmic transformation of ICV as covariates, and is presented in Fig. 3. The model indicated a statistically significant non-linear increase in risk of glioma with increasing ICV above 1394 ml (*p* < 0.001). However, the model also indicated an increase in risk of low-grade glioma with ICV on the lowest ends of the scale (< 1394 ml). In total, only 16 of 154 cases (10.4%) had an ICV below this threshold.

In Table 1, mean ICV in different molecular subtypes are presented (non-specified diffuse gliomas are excluded, n = 17). As seen, mean ICV in females with oligodendroglioma was 125 ml larger than in females with astrocytoma (*p* < 0.001). In males, there was no statistically significant difference in mean ICV between cases with oligodendrogliomas and astrocytomas (*p* = 0.726).

**Table 1:** Intracranial volumes in astrocytomas vs. oligodendrogliomas

	Females			Males		
	Astrocytoma (n = 29)	Oligodendro-glioma (n = 31)	*p*-value*	Astrocytoma (n = 33)	Oligodendro-glioma (n = 44)	*p*-value*
ICV, ml (mean ± SD)	1453 ± 102	1578 ± 153	** < 0.001**	1729 ± 156	1717 ± 138	0.726

Bold text indicates statistical significance

*Independent samples t-test

## Discussion

In this multicenter case–control study based on population-based data, we found a non-linear relationship between ICV and risk of *IDH*-mutant low-grade glioma, with increased risk both above and below a threshold in intracranial volume. Although our study should be replicated by others, a relationship between ICV and risk of glioma seems likely.

Several other studies have found associations between body or organ size and risk of cancer, including lymphomas, leukemia, gastric cancer, testicular cancer, malignant melanoma, cervical cancer, colorectal cancer and breast cancer [12]. Indeed, a positive association between height, which is correlated to brain size [13, 14], has been reported for risk of primary brain tumors in adults [15–17]. It is therefore not surprising that also ICV is associated with glioma risk, like we previously observed in high-grade gliomas [4].

The nature of the relationship between ICV and risk of *IDH*-mutant low-grade glioma appears to be non-linear, and the binomial logistic regression suggested higher risk both below and above an ICV threshold. However, as the large majority of both cases and controls in this study had ICV above the statistical threshold of lowest risk in the regression analyses, our results are mainly in line with our previous findings on the association between ICV and high-grade glioma [4]. A much-debated study on cancer etiology suggested that most cancers are caused by “bad luck” since risk of cancer in general is correlated to the lifetime number of stem cell divisions [18]. Although the number of glial cells in relation to ICV is not known, another study on our control population found that the relative size of white matter increases with increasing ICV [19]. Still, the possible increase in risk of low-grade glioma in the lower-end ICV cases in our data may not be clearly explained by number of cells at risk. The “bad luck” hypothesis has been criticized, and an evolutionary multistage model of carcinogenesis has been suggested to better explain findings in the same dataset [20]. This model may better reflect cancer etiology as a function of random DNA replication errors and Darwinian fitness, and although it does not directly explain our findings of increased risk in lower-end ICV cases, it suggests that the etiology of cancer is more complex than just random mutations. Genome-wide association studies have identified single nucleotide polymorphisms (SNP) associated with risk of glioma [21]. Further, statistical models for life-time glioma risk and prediction of glioma subtype based on SNPs, age and sex have been developed [22]. Different SNPs may possibly affect glioma risk both directly and indirectly through effects on ICV, though any such effects are currently undetermined. Another explanation is that our u-shaped statistical finding was based on rather few cases with small ICV and could be a result of overfitting.

In both the present data from low-grade glioma and our previous study in high-grade glioma, the epidemiologically well-documented higher risk of glioma in men [5] was no longer evident after adjustment for ICV. If there is a link between organ size and lifetime number of stem cell divisions at risk of cancer [18], a reduced effect of sex on glioma risk after adjustment for ICV is expected since males in general have larger brains than females [3].

While we observed a linear relationship between ICV and risk of high-grade glioma [4], the current data suggests that the relationship may be non-linear for *IDH*-mutant low-grade glioma. Although this should be replicated by others, the impact of ICV as a potential risk factor may be different in low- and high-grade gliomas, perhaps reflected in the different age-specific incidence rates [6]. Since low-grade gliomas occur earlier, different mechanisms can be involved, and the potential relationship may also differ in molecular subgroups. For example, WHO grade 2 oligodendroglioma incidence is reported to be higher in males with an overall male-to-female ratio of 1.25, while the male-to-female ratio for glioblastoma is 1.34 [6]. Further, it has been reported that stem cell division rates decrease with high age, possibly explaining reduced risk of cancer in very old humans for certain types of malignancies [23]. Although subgroups are small and should be interpreted with caution, mean ICV was larger in females with oligodendroglioma compared to females with astrocytoma, while there was no statistically significant difference in ICV between molecular subtypes in males. In a study estimating number of glial cells in human brains, a slightly higher proportion of neocortical oligodendrocytes to astrocytes ratio in females compared to males was found [3]. However, the sample size was small and glial cell estimation is notoriously difficult [24]. As the ratio between oligodendrocytes and astrocytes in males and females in the entire brain is poorly mapped and possible sex-specific differences in this ratio with varying ICV is unknown, disentangling possible sex-specific relationships between glioma subtypes and ICV is highly complex.

### Strengths and limitations

Strengths of the present study are the large, population-based glioma cohort from three different centers, and the large, representative population-based control group. This eliminates the risk of referral bias, and the external validity should be high. In addition, the use of a refined automatic segmentation method for ICV eliminates the risk of observer-dependent assessment bias. Since accurate assessment of brain volume is impossible in glioma patients due to the infiltrative tumor growth, ICV was used as a proxy for lifetime maximum brain volume. Brain volume and ICV are found to be highly correlated, and ICV does neither change significantly after the age of 16 nor decline significantly with aging [25]. Further, as median age in the case population was lower than in the control population, life-time radiation exposure is highest in the control population. Consequently, the observed age difference in cases and controls is not likely to affect our results. A limitation of this study is that various MRI scanners were used in the glioma population, and we did not have scanner id in all patients. However, in our previous study the variance of the MRI-scanner (scanner id) was estimated at zero [4], and it is reasonable to assume that the results would have been similar in the present study. Another limitation is the lack of biological exploration in our study, such as possible biomarkers for stem cell division rates or other objective evidence to confirm ICV as a risk factor for glioma tumorigenesis.

## Conclusions

Intracranial volume seems to be risk factor for *IDH*-mutant low-grade glioma, but the relationship may be non-linear with increased risk both above and below a specific threshold in intracranial volume.

## Data Availability

The dataset analysed during the current study are available from the corresponding author on reasonable request.
